# IL28B Polymorphisms and Clinical Implications for Hepatitis C Virus Infection in Uzbekistan

**DOI:** 10.1371/journal.pone.0093011

**Published:** 2014-03-24

**Authors:** Dinara Khudayberganova, Masaya Sugiyama, Naohiko Masaki, Nao Nishida, Motokazu Mukaide, Dildora Sekler, Renat Latipov, Kan Nataliya, Suyarkulova Dildora, Said Sharapov, Guzal Usmanova, Mahmarajab Raxmanov, Erkin Musabaev, Masashi Mizokami

**Affiliations:** 1 Department of Hepatic Diseases, The Research Center for Hepatitis and Immunology, National Center for Global Health and Medicine, Ichikawa, Japan; 2 Institute of Virology, Ministry of Public Health of Uzbekistan, Tashkent, Uzbekistan; Harvard Medical School, United States of America

## Abstract

**Aims:**

Genome-wide association studies highlighted single nucleotide polymorphisms (SNPs) within the IFNL3/IL28B locus predict the treatment outcome for patients with HCV. Furthermore, SNPs in newly discovered IFNL4 are shown to have population-specific correlation with spontaneous clearance of HCV. The aim of this study was to examine the prevalence and clinical significance of the outlined SNPs in a population from Central Asia, a multi-ethnic region with a developing economy and a high prevalence of HCV infection.

**Methods:**

One hundred and thirty-five chronic HCV patients from Uzbekistan were enrolled. DNA specimens were extracted from peripheral blood mononuclear cells and the IFNL3 SNPs (rs8099917, rs12979860) were genotyped by the Invader Plus assay, the TaqMan assay, and by direct sequence analysis. The IFL4 region (ss469415590) was sequenced.

**Results:**

Of the 135 patients that completed 24 or 48 weeks of treatment with Peg-IFN-α plus RBV, 87.4% were of Central Asian (CA) ancestry and 12.6% were of Eastern European (EE) ancestry. A non-virological response was observed in 21.2% of CA and in 35.3% of EE, respectively (p<0.32). The rs12979860 was strongly associated with treatment response (OR, 5.2; 95% CI, 1.9–14.6; p<0.004) in the overall sample; however, SNP rs8099917 was the most predictive of outcome for CA group (OR, 6.9; 95% CI, 2.6–18.0; p<0.002). The allele frequency of IFNL4 SNP, ss469415590, was identical with that of rs12979860 in all samples.

**Conclusions:**

SNPs in IFNL3 and IFNL4 can be used to predict HCV treatment outcome in a population of Central Asian ancestry.

## Introduction

Chronic Hepatitis C virus (HCV) infection is a global healthcare problem, with thestimated number of people positive for anti-hepatitis C virus antibodies increasing from >122 million to >185 million between 1990 and 2005 [1]. Central and Eastern Asia, North Africa, and the Middle East are thought to have the highest prevalence of anti-HCV antibodies (>3.5%) [1]. Although successful implementation of direct-acting antiviral therapy was recently reported in Western countries, combined treatment with pegylated interferon-alpha (PEG-IFN-α) plus ribavirin (RBV) is still the most effective treatment for patients with chronic hepatitis C in central Asia [2], [3]. However, this treatment is both costly and associated with significant adverse side effects, resulting in poor compliance. Furthermore, approximately half of treated patients fail to achieve a sustained virological response (SVR). Of the various host (age, sex, race, fibrosis stage) and viral (genotype, viral load) factors (reviewed in ref 2, 4) associated with the effectiveness of IFN-based therapy, the recently discovered genetic polymorphisms (SNPs) of *interleukin 28B (IL28B)* has been reported. The SNPs had the most significant predictive value for treatment outcomes in several countries [4]–[6]. The polymorphism in *IL28B* forms a cluster of single nucleotide polymorphisms (SNPs) that appear to delineate a genetic haplotype within a very low recombination fragment containing the *IL28B* gene. Among all the SNPs within this cluster, rs12979860 and rs8099917 are the strongest markers of the haplotype, and consistently predict treatment outcomes for patients receiving IFN-based regimens [7]–[10]. Recently, a study examining a cohort of African Americans identified a novel *interferon lambda 4 (IFNL4)* gene located in an immediate proximity to the *IL28B*, and suggested that it was associated with HCV clearance [11]. The IFNL4 SNP improved the prediction rate of IFN-based regimens in African Americans, and more recently in Caucasians and Japanese [12]–[20].

Uzbekistan is one of the most populous countries in Central Asia. The HCV infection prevalence in the general population is very high, at >6.4%, and is >20% in “high-risk” groups [21]. The most prevalent HCV genotypes are HCV-1b followed by 3a [22], [23]. Because the population of Uzbekistan comprises individuals from many different genetic backgrounds, the aim of this study was to examine the prevalence and clinical relevance of IL28B and IFNL4 polymorphisms in the context of the ethnic ancestry background of populations in this country.

## Methods

### Study population

Outpatients with chronic HCV infection treated with PEG-IFN-α plus RBV at the Institute of Virology Ministry of Public Health of Uzbekistan between May, 2009 and December, 2011 were enrolled in the study. The study protocol was approved by the Institutional Review Board and Institute of Virology Ministry of Public Health of Uzbekistan. Written informed consent was obtained from all patients. This study conforms to the provisions of the Declaration of Helsinki (as revised in Seoul, Korea, October 2008). The patients and their physicians completed a written questionnaire, which was used to collect socioeconomic, demographic, clinical, and laboratory data. The data were then subjected to a per-protocol analysis. The diagnosis of HCV infection was based on the detection of anti-HCV antibodies. The viral load was determined using the AmpliSens HCV-Monitor-FL (InterLabService Ltd., Moscow) Real-Time PCR kit, which has a detection range limits of 300–10^8^ IU/mL (equivalent to 1×10^3^–3×10^8^, HCV RNA copies/mL).

Patients consisting of 135 subjects received the full treatment course (see below). Data derived from the patients that received at least 80% of the prescribed drug dose were used for the outcome association study. Patients with end-stage kidney disease, hepatocellular carcinoma, or decompensated liver cirrhosis (as defined by a Child-Pugh score greater than 6) were excluded. The ethnic background of each individual was assessed according to the patient questionnaire; patients of Uzbek, Kyrgyz, Kazakh and Tajik ethnicities were included into the Central Asian ancestry (CA) group, patients of Russian and Tatar ethnicities were included into the Eastern Europe (EE) group Other ethnic minorities were excluded from the study.

### Treatment for hepatitis C

Patients were treated with a weekly dose of PEG-IFN-α (1.5 mcg/kg) coupled with a daily dose RBV (1000 mg/day for patients up to 75 kg, and 1,250 mg/day for those over 75 kg). The viral load was determined by real-time reverse transcription-polymerase chain reaction (RT-PCR) prior to the start of treatment. On-treatment viral kinetics were evaluated at Weeks 4 and 12. To evaluate the power of the SNP genotype as a predictor of responses to antiviral treatment, all patients were classified into one of two groups: (I) non-responders (including those who still had detectable HCV RNA levels at Weeks 4 and 12 or at the post-treatment follow-up [24 weeks after treatment]); and (II) responders (including those with no detectable HCV RNA both during and/or after treatment).

Treatment was stopped if a patient failed to achieve a 2log (or greater) reduction in viral load after 12 weeks.

### IL28B genotyping

Whole blood was collected from all participants and centrifuged to separate the buffy coat. Genomic DNA was extracted from the buffy coat (containing peripheral blood mononuclear cells) using a QIAamp DNA Mini Kit (QIAGEN, Venlo, Netherlands).

All patients were genotyped for the SNPs rs8099917, rs12979860, rs8103142, and rs11881222 using a probe-based assay as previously described [24]. Two different probe-based assays, Invader Plus and the TaqMan probe assay, were used, and their sensitivity and specificity were compared with those of direct sequencing. For direct sequencing, the region of genomic DNA around rs12979860 was amplified using primers t63_L (5′-GGAAGGAGCAGTTGCG-3′), t63_R (5′-GGCTGTGGGTCCTGT-3′), t64_L (5′-GACAGGAACGGGTGTATG-3′), and t64_R (5′-AGCTCTGATGTTGGGAAAG-3′).

### Statistical analysis

Data were analyzed using SPSS 17.0 (SPSS for Windows, Chicago, IL). Categorical variables were expressed as numbers and percentages and continuous variables with a normal distribution were expressed as the mean and standard deviation. The Chi-squared and Fisher's exact tests were used where appropriate, and p<0.05 was considered statistically significant. Statistical odds ratios (OR) for treatment prediction were derived by logistic regression analysis.

## Results

### Comparison of the genotyping assays

Genotyping of *IL28B* was performed using the Invader Plus and TaqMan probe-based assays [24], and by direct sequencing. There was 100% concordance between the two assays, and there was 99.2% agreement between the two assays and direct sequencing (i.e., a discrepancy of 0.8%) (**Table 1**). Therefore, we used the broadly-prevalent TaqMan probe assay to examine the association between SNPs and treatment responses in the present study.

**Table 1:** Summary of results of genotyping by three different methods.

Total n = 135		No.(%) of cases with genotype by:			
SNP	Genotype	Direct sequencing	Invader	TaqMan	Concordance
**rs12979860**	**CC**	**57**	**57**	**57**	
	**CT**	**64**	**64**	**64**	**1**
	**TT**	**14**	**14**	**14**	
**rs8099917**	**TT**	**90**	**89**	**89**	
	**TG**	**40**	**40**	**40**	**0.992**
	**GG**	**5**	**6**	**6**	

### Association between SNPs and treatment responses

**Figure 1:**
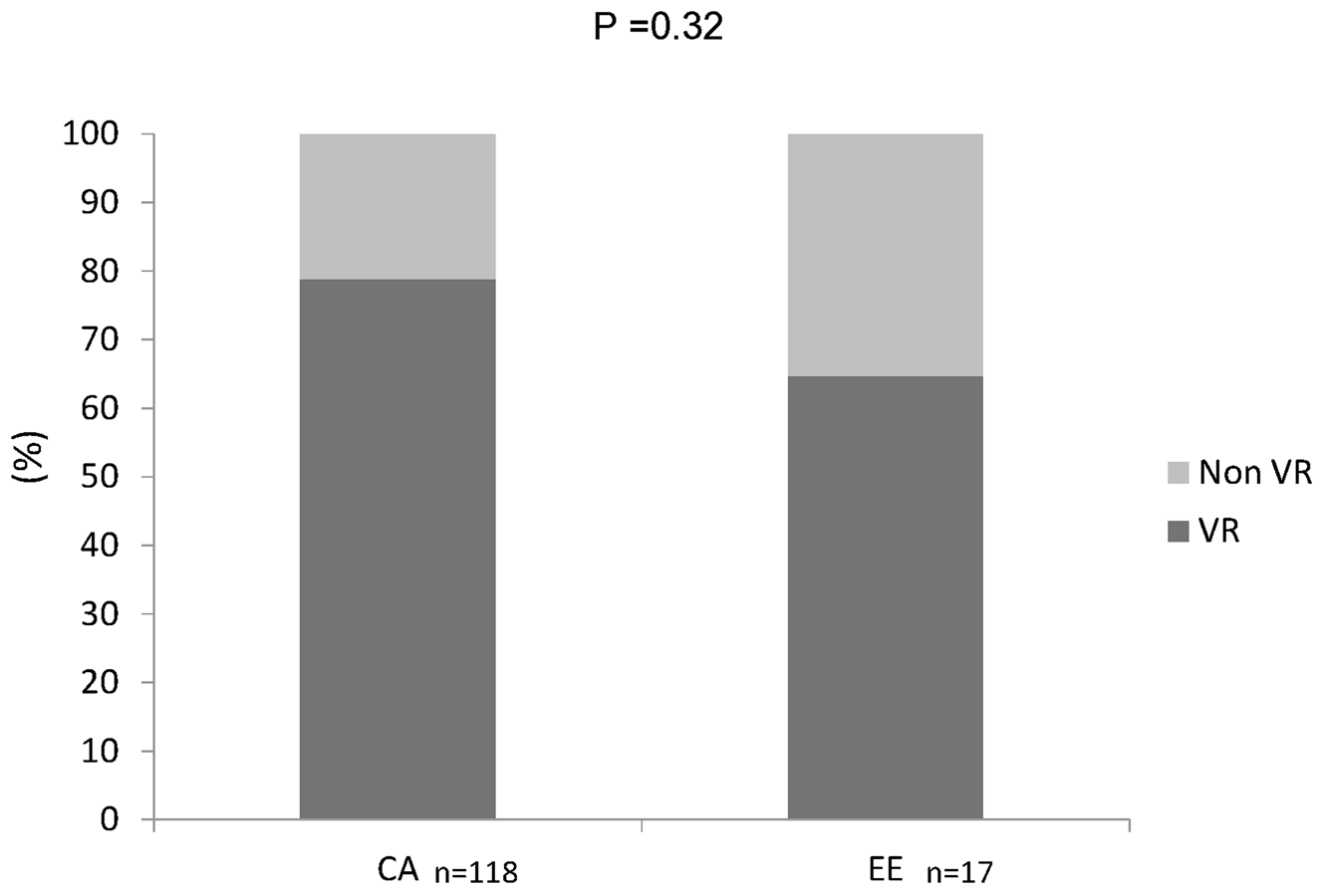
Different HCV treatment outcomes in groups of individuals of central Asian (CA) or eastern European (EE) ancestry. Treatment outcome was measured in terms of virological and non-virological response (VR and NVR, respectively) (see text for details).

The characteristics of each patient group are summarized in Table 2. One hundred thirty five patients (87.5% CA, 12.5% EE) completed either 24 or 48 weeks of treatment with Peg-IFN-α plus RBV. There was no significant difference between the groups in terms of age, gender, HCV viral load, and viral genotype (**Table 2**). There was no statistically significant difference between the percentages of CA and EE that showed a NVR (21.2% and 35.2%, respectively; p<0.32) (**Fig. 1**). However, there was a significant difference in the prevalence of SNPs within *IL28B* and *IFNL4* between VR and NVR in each ethnic. To evaluate the clinical applicability of individual SNPs, we calculated the predictive ORs for each SNP between VR and NVR in each ethnic (**Table 3**). All of the identified SNPs (favorable genotype) predicted positive response to treatment outcome in the overall study population and in the CA population, but not in the EE population. Interestingly, the polymorphism identified in the newly discovered *IFNL4* gene, ss469415590 [11], showed a strong linkage with the rs12979860 SNP around *IFNL3* in the overall study population; therefore, each had equal predictive value (**Table 3**). The most informative marker to predict VR of HCV treatment outcome was rs8099917 (OR, 5.75; 95% CI, 2.4–13.6, p<0.001), followed by rs12979860/ss469415590 (OR, 5.2;95% CI, 1.9–14.6; p = 0.002). The predictive values of the SNPs are shown here for the entire studied population inclusive all HCV genotypes. There was no significant difference in predictive power (OR) of the SNPs when population was analyzed in the context of different HCV genotypes (1 vs non-1), however statistical power of the analysis was lower, most likely due to the smaller size of the non-1 genotype infected patients in this study.

**Table 2:** Summary of population completed antiviral treatment for chronic HCV.

	Central Asian			East European			Overall		
	VR	NVR	p	VR	NVR	p	VR	NVR	p
**n.**	**93**	**25**		**11**	**6**		**104**	**31**	
**Age (mean years old ± SE)**	**39.7±1.5**	**39.7±3.2**	**0.437**	**45.2±3.6**	**39.3±5**	**0.358**	**40.6±1.3**	**37.9±2.9**	**0.346**
**Baseline HCV viral load (mean ×106±SE)**	**3.1±8.9**	**4.2±2.3**	**0.609**	**1.6±6**	**1.2±5**	**0.663**	**2.9±6.7**	**3.4±1.7**	**0.727**
**HCV genotype (1/non-1)**	**77/16**	**22/3**	**0.759**	**11**	**6**		**88/16**	**28/3**	**0.673**
**Treatment duration (mean months±SE)**	**7.6±0.2**	**8±0.5**		**8.3±0.8**	**6.0±1.2**		**7.7±0.5**	**7.6±0.8**	
**Drug configuration (IFN/Peg IFN)**	**38/55**	**14/11**	**0.176**	**7/4**	**5/1**	**0.6**	**59/45**	**12/19**	**0.101**
**IL28B (rs8099917)**									
** MA: n(%)**	**71(76.4)**	**8(32)**	**<0.001**	**8(72.7)**	**3(50)**	**0.6**	**79 (76)**	**11 (35.5)**	**<0.001**
** HE&MI: n(%)**	**22(23.6)**	**17(68)**		**3(27.3)**	**3(50)**		**25 (24)**	**20 (64.5)**	
**IL28B (rs12979860)**									
** MA: n(%)**	**47(50.5)**	**4(16)**	**0.003**	**5(45.5)**	**1(16.6)**	**0.333**	**52 (50)**	**5 (16.1)**	**<0.001**
** HE&MI: n(%)**	**46(49.5)**	**21(84)**		**6(54.5)**	**5(83.4)**		**52 (50)**	**26 (83.9)**	
**IFNL4 (ss469415590)**									
** MA: n(%)**	**47(50.5)**	**4(16)**	**0.003**	**5(45.5)**	**1(16.6)**	**0.333**	**52 (50)**	**5 (16.1)**	**<0.001**
** HE&MI: n(%)**	**46(49.5)**	**21(84)**		**6(54.5)**	**5(83.4)**		**52 (50)**	**26 (83.9)**	

**Table 3:** SNPs showed statistical significance in predicting treatment outcome in studied population.

Ethnic origin	ss469415590 TT	rs8099917 TT	rs12979860 CC
	OR (95% CI)	OR (95% CI)	OR (95% CI)
**Central Asian**	5.364 (1.7–16.8) *	6.858 (2.6–18.0) *	5.364 (1.7–16.8) *
**East EU**	4.167 (0.4–48.4)	2.667 (0.3–21.3)	4.167 (0.4–48.4)
**Overall**	5.2 (1.9–14.6) *	5.745 (2.4–13.6) *	5.2 (1.9–14.6) *

*(p≤0.05).

### Genetic differences between ethnic groups

**Figure 2:**
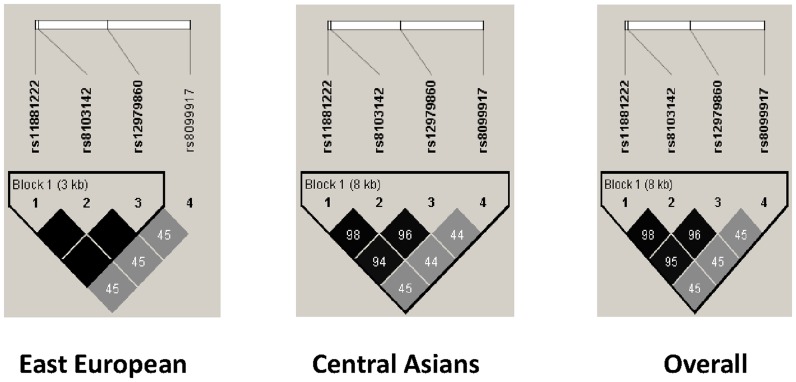
Linkage disequilibrium diagram showing clustering of the studied SNPs. The diagram was generated using HaploView software (available through the HapMap project).

**Figure 3:**
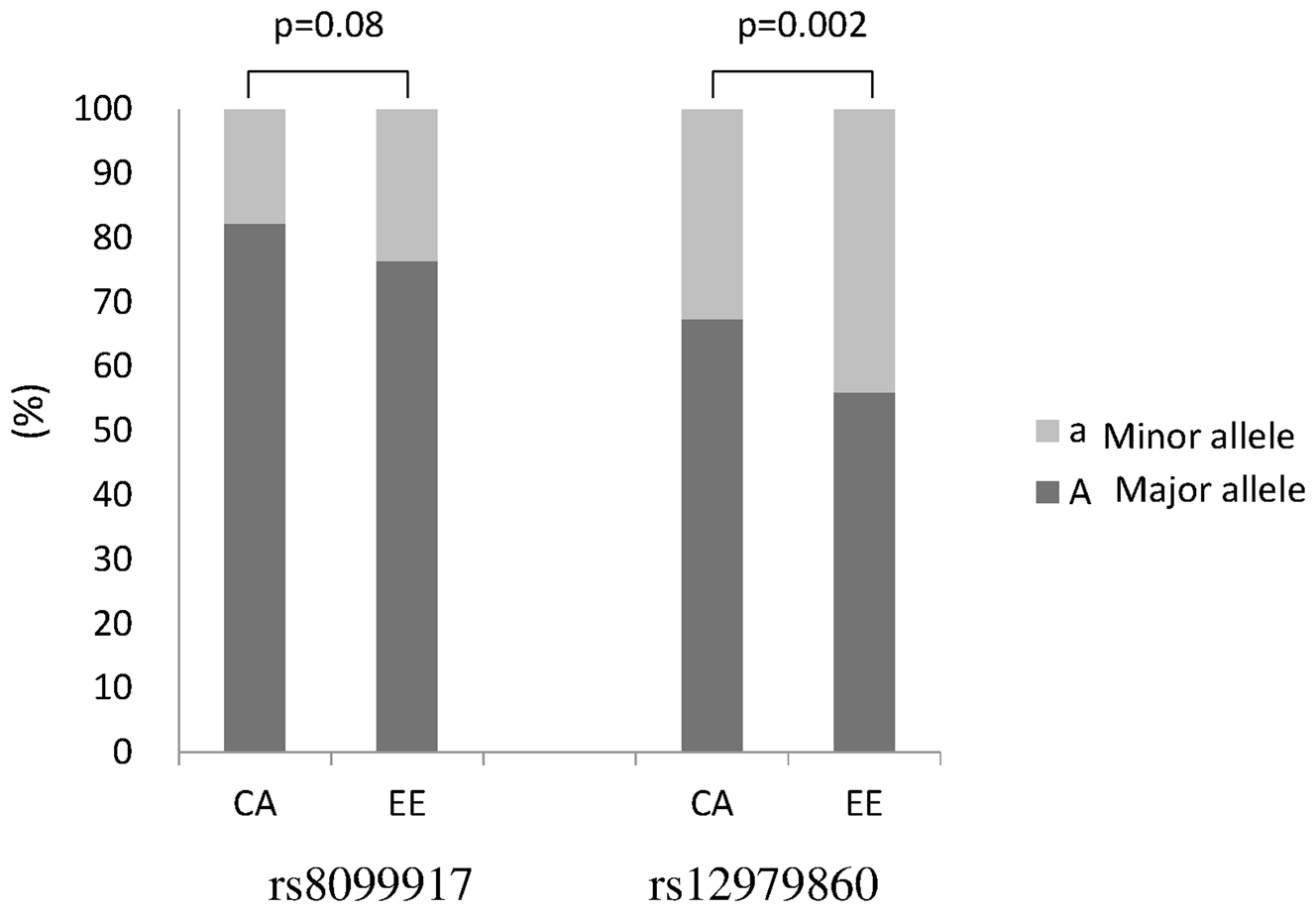
Allele frequencies of the tested SNPs (n = 135). The capital letter “A” represents ancestral (“major”) alleles and lower case letter “a” represents mutant alleles (“minor”). CA: population with central Asian ancestry; EE: population with eastern European ancestry.

The alleles associated with all of the tested SNPs were in the Hardy-Weinberg equilibrium. The rs12979860, rs8103142, and rs1188122 SNPs showed high linkage disequilibrium (LD) in both ethnic groups (**Fig. 2**). There was no difference in the frequency of rs8099917 alleles between the CA and EE populations; however, a minor allele of the rs12979860 SNP was observed more frequently in the EE group (0.32 *vs.* 0.44; p<0.002) (**Fig. 3**). The rs8099917 SNP had a higher predictive value than rs12979860/ss469415590 in the CA population (**Table 3**), whereas the reverse tended to be true in the EE population, although the differences were not statistically significant.

## Discussion

The aim of this study was to examine the prevalence and clinical significance of SNPs within the *IFNL3/IL28B* and *IFNL4* alleles in a population of HCV-infected patients in Central Asia. We also evaluated the ability of these SNPs to predict responses to anti-HCV treatments in this population. We found that rs12979860 and rs8099917 were informative markers of treatment response in Uzbekistan with different ethnicity. The rs8099917 genotype TT was the most common in the overall study population (67.8%), followed by the rs12979860 genotype CC (49%). This is the first report showing the distribution and linkage between the recently described *IFNL4* ss469415590 SNP [11] and the *IL28B* rs12979860 SNP in HCV-infected individuals in Uzbekistan.

According to the Human Haplotype Mapping project, only 15–19% of Caucasians carry the rs8099917 G allele. Notably, the GG genotype of rs8099917 was identified in 3.6% of patients in the present study, a lower prevalence than that observed in other countries [7]–[10]. These results agree with those of a previous study showing the variability of allele frequencies (2–31%) between different ethnic groups [25].

The rs8099917 SNP was a better predictor of treatment outcome in subjects of CA ancestry than the rs12979860/ss469415590 SNPs (**Table 3**). However, the reverse tended to be true for patients with EE ancestry. This is in agreement with the results of a previous study that examined populations of Western European ancestry [7], [10]. A greater number of individuals of Eastern European ancestry must be examined to confirm the trend observed in the present study. Finally, although previous reports show that combined polymorphisms may show increased predictive value in terms of a SVR [26], no significant improvements were noted for the populations examined herein. Interestingly, the degree of LD between rs12979860 and the two SNPs within the IL28-encoding gene identified herein was slightly different in the two populations studied (Fig. 2), i.e., a strong LD was observed among patients of EE ancestry. One possible explanation for this is the smaller size of this patient population. Thus, we need to confirm our findings in a larger cohort. Another interesting observation is that, differently from a previously described Japanese population [9], we found a very low LD between rs8099917 and SNPs within the IL28B-encoding region; nevertheless both the favorable genotype of rs12979860 and rs8099917 were independent predictors of treatment outcome, suggesting the possibility of different mechanisms of involvement of the genetic regions around IL28B.

Predictive power of the SNPs, particularly of the IFNL4 ss469415590 variation reported here was in the range of that reported among Caucasians with HCV/1b [14], [15]. The fact that predictive power of genetic markers ranges vastly across different reports even within a highly homologous genetically population as Japanese (OR from 4.7 to 19.5) [19], [20], reinforces importance of replication and meta-analyses of such investigations across and within populations with different ethnic background.

In conclusion, genotyping of *IL28B* locus polymorphisms could help to predict responses to PEG-IFN-α plus RBV therapy in a Central Asian population. As protease inhibitors gain popularity as a form of HCV therapy, the clinical application of *IL28B* genotyping to this population may help to identify patients who might benefit from therapies other than triple therapy. Thus, genotyping the rs12979860/rs8099917 polymorphisms are still the best known markers that could be used to predict patients' responses to IFN/RBV before initiation of the treatment. This can be important marker for the choice of individually tailored anti-HCV therapy.
